# Green Ethanolic Repercolation of *Larix decidua* Needles: Phytochemical Profiling and In Vivo Modulation of the Oxidative–Nitrosative Axis in Acute Sterile Inflammation

**DOI:** 10.3390/nu18030538

**Published:** 2026-02-05

**Authors:** Dinu Bolunduț, Alina Elena Pârvu, Cristina Moldovan, Florica Ranga, Marcel Pârvu, Ciprian Ovidiu Dalai, Mădălina Țicolea, Andra Diana Cecan, Raluca Maria Pop

**Affiliations:** 1 Department of Morphofunctional Sciences, Faculty of Medicine, “Iuliu Haţieganu” University of Medicine and Pharmacy, 400012 Cluj-Napoca, Romania; bolundut_dinu@elearn.umfcluj.ro (D.B.); parvualinaelena@umfcluj.ro (A.E.P.); madalina.ticolea@umfcluj.ro (M.Ț.); andra.cecan@umfcluj.ro (A.D.C.); 2 Department of Medical Oncology, “Ion Chiricuță” Institute of Oncology, 400015 Cluj-Napoca, Romania; 3 Department of Pharmaceutical Chemistry, Faculty of Pharmacy, “Iuliu Hațieganu” University of Medicine and Pharmacy, 400012 Cluj-Napoca, Romania; 4 Food Science and Technology, Department of Food Science, University of Agricultural Science and Veterinary Medicine Cluj-Napoca, 400372 Cluj-Napoca, Romania; florica.ranga@usamvcluj.ro; 5 Department of Biology, “Babeș Bolyai” University, 400015 Cluj-Napoca, Romania; marcel.parvu@ubbcluj.ro; 6 Department of Surgical Sciences, Faculty of Medicine and Pharmacy, University of Oradea, 410087 Oradea, Romania; cipridalai@gmail.com; 7 Pharmacology, Toxicology and Clinical Pharmacology, Department 2—Functional Sciences, Faculty of Medicine, “Iuliu Haţieganu” University of Medicine and Pharmacy, 400012 Cluj-Napoca, Romania; raluca.pop@umfcluj.ro

**Keywords:** phytochemicals, inflammation, antioxidants, oxidative stress

## Abstract

**Background/Objectives**: *Larix decidua* has been used in traditional medicine for the treatment of various inflammatory conditions. Although their use has been recognized in alternative medicine, the scientific documentation of the antioxidant and anti-inflammatory potential of ethanolic extracts from its needles remains insufficiently characterized. The present study aimed to characterize the phytochemical profile of the ethanolic *L. decidua* extract, evaluate its in vitro antioxidant capacity, and investigate its therapeutic and prophylactic effects on oxidative–nitrosative stress and inflammation. **Methods***: L. decidua* needles were extracted using a modified Squibb repercolation method. Polyphenol and flavonoid content were quantified, and individual phenols were identified by HPLC-DAD-ESI^+^. The in vitro antioxidant activity was evaluated using DPPH, FRAP, H_2_O_2_, and NO scavenging assays. The therapeutic and prophylactic in vivo potential was evaluated in a model of acute inflammation induced with turpentine in male Wistar rats. Serum oxidative markers (TOS, TAC, OSI, MDA, AOPP, 8-OHdG, NO, 3-NT, SH) and inflammatory markers (NFκB-p65, IL-1β, IL-18) were quantified. **Results**: The extract contained high levels of flavonols and hydroxybenzoic acids; kaempferol glycosides and catechin were the dominant constituents. In vitro, the extract exhibited radical scavenging activities. In vivo, *L. decidua* attenuated oxidative and nitrosative stress, restored antioxidant defense, and reduced NFκB-p65, IL-1β, and IL-18 levels in a concentration-dependent manner. The L100 concentration most closely approximated the values produced by Trolox and diclofenac. **Conclusions**: The ethanolic *Larix decidua* needle extract exerted antioxidant and anti-inflammatory effects in a rat model of acute sterile inflammation, attenuating systemic oxidative–nitrosative stress and pro-inflammatory mediators in a concentration-dependent manner. These preclinical findings support further investigation of standardized *L. decidua* needle preparations as polyphenol-rich nutraceutical/functional ingredient candidates within preventive and adjunct nutrition strategies targeting oxidative stress-driven inflammation.

## 1. Introduction

In traditional European medicine, conifer extracts have been important therapeutic tools for centuries. They have been used to manage inflammation, accelerate wound healing, and treat various types of infections. Traditional Austrian and Balkan communities used balsams and resins from *Larix decidua*, *Picea abies*, and *Pinus nigra* as topical treatments for wounds, ulcers, and rheumatic pain [1]. Historical medical observations have been confirmed by in vitro and in vivo studies. These studies highlight active compounds in conifer extracts, such as diterpenoids, lignans, and hydroxycinnamic acids. Scientific studies on *Larix decidua* and its extracts have shown beneficial results. In vivo reepithelization, demonstrated by keratinocyte scratch assays, supports the theory of wound healing potential [2]. Also, from a historical point of view, in traditional European medicine, extracts obtained from various types of pine were used for the treatment of respiratory or rheumatic diseases. The evidence obtained from contemporary experiments supports the historical observations, demonstrating antioxidant, antiseptic, analgesic, decongestant effects in various in vitro experiments [3]. Beyond ethnomedicinal relevance, *L. decidua* preparations in their various forms have been the subject of multiple investigations in nutrition-related contexts. The observed results tend to favor their use as sources of functional ingredients rather than as conventional foods. In an applied livestock production setting, supplementation with *Larix decidua* L. sawdust was evaluated as a feed additive for dairy cows, with monitoring of blood and milk parameters, and reported good palatability, suggesting the feasibility of developing feed supplements [4]. In addition, a water-soluble polysaccharide found in abundance in the heartwood of larch species, arabinogalactan, has been the subject of several studies investigating its use as a food supplement with a dietary fiber role and/or as a food additive. It has been described as having a favorable safety profile based on toxicity studies in accordance with current regulations [5]. Taken together, all these aspects support the rationale for studying *L. decidua* extract in order to obtain scientific evidence that may facilitate its standardization and subsequent administration as a nutraceutical ingredient for the modulation of oxidative stress and inflammation, central mechanisms in a range of chronic pathologies.

To assess the systemic oxidative–nitrosative imbalance associated with acute sterile inflammation, we quantified integrative redox indices (TOS, TAC, and OSI), molecular markers of damage (MDA, AOPP, and 8-OHdG), biomarkers of nitrosative stress (NO and 3-NT), and thiol status (SH), along with key mediators of inflammation (NFκB-p65, IL-1β, and IL-18) [6,7,8,9,10,11,12,13]. Since oxidative stress and inflammation are mutually reinforcing, modulating this oxidative–inflammatory loop by bioactive phytochemicals is a plausible strategy not only to attenuate acute injury but also to reduce the risk of progression to chronic inflammatory states [14].

In the present study, we selected *Larix decidua* needles because they represent a renewable, non-destructive, and widely available botanical matrix that can be processed using food-grade ethanol via a green repercolation approach, facilitating future standardization and potential nutraceutical development. The aims of study were first to perform a phytochemical analysis and an in vitro antioxidant activity evaluation of an ethanolic extract of fresh *Larix decidua* needles, and afterwards to do an in vivo assessment of the antioxidant and anti-inflammatory potential of the same extract on a turpentine oil-induced acute inflammation rat model. To the best of our knowledge, this is the first study to integrate a green ethanolic repercolation approach with comprehensive phytochemical profiling and the parallel evaluation of both therapeutic and prophylactic in vivo effects of *Larix decidua* needle extract in a model of acute sterile inflammation.

## 2. Materials and Methods

### 2.1. Plant Material and Extract Preparation

*Larix decidua Mill.* plants were obtained from the “Alexandru Borza” Botanical Garden Cluj-Napoca, Romania. Plants were identified and voucher specimens (No. 674531/2023) were deposited at the Herbarium of “Alexandru Borza” Botanical Garden Cluj-Napoca, Romania. Larch needles freshly collected were cut into fragments of 0.5–1.0 cm, and extracted with 70% ethanol (Merck, Bucuresti, Romania) in the Mycology Laboratory of “Babes-Bolyai” University, Cluj-Napoca, by a modified Squibb repercolation method, producing a 1:1 (*w*/*v*) solution [14].

### 2.2. Phytochemical Analysis

#### 2.2.1. Determination of Total Polyphenol Content (TPC)

The TPC in *Larix decidua* needle extract were quantified using a Folin–Ciocalteu assay with minor adjustments. The reaction was initiated by combining 2 mL of extract with 1 mL Folin–Ciocalteu reagent and keeping the mixture protected from light for 5 min. The mixture was then brought to a final volume of 25 mL with sodium bicarbonate solution (290 g/L) and incubated for 60 min at room temperature. Absorbance was read at 760 nm using a Jasco V-530 UV–Vis spectrophotometer (Jasco International Co., Ltd., Tokyo, Japan). Quantification was based on a gallic acid calibration curve, and results were expressed as mg gallic acid equivalents per g dry extract (mg GAE/g d.w.) [15].

#### 2.2.2. Determination of Total Flavonoid Content (TFC)

The TFC were assessed by an AlCl_3_-based colorimetric method. Absorbance was measured at 510 nm, and quercetin was used for external calibration; data were reported as mg quercetin equivalents per g dry extract (mg QE/g d.w.) [16]. Both TPC and TFC analyses were determined in triplicate.

### 2.3. HPLC-DAD-ESI^+^ Analysis of Phenolic Compounds

The phenolic profile of *Larix decidua* extract was analyzed using an Agilent 1200 HPLC system (Agilent Technologies, Santa Clara, CA, USA) equipped with a quaternary pump, a solvent degasser, an autosampler, a diode array detector (DAD), and a mass spectrometer (MS) equipped with a single mass spectrometer (MS) Kinetex XB-C18 column (4.6 × 150 mm, 5 μm; Phenomenex, Torrance, CA, USA) maintained at 25 °C.

The mobile phase consisted of (A) water with 0.1% acetic acid and (B) acetonitrile with 0.1% acetic acid, administered at 0.5 mL/min under the following gradient (expressed as %B): 0 min, 5%; 0–2 min, 5%; 2–18 min, 5–40%; 18–20 min, 40–90%; 20–24 min, 90%; 24–25 min, 90–5%; 25–30 min, 5%. UV-Vis spectra were collected in the range of 200–600 nm, and chromatograms were monitored at 280 and 340 nm.

Mass spectrometry detection was performed in positive electrospray ionization (ESI^+^) mode with the following settings: capillary voltage, 3000 V; source temperature, 350 °C; nitrogen flow, 7 L/min; and full scan from *m*/*z* 120–1200. Data acquisition and processing were performed using Agilent ChemStation software, version B.02.01-SR2.

Phenolic compounds were identified by matching retention times, UV-Vis spectra, and mass spectra to those of reference standards, the literature data, and databases such as Phenol-Explorer.

Calibration curves were generated for the quantification of phenolic compounds by injecting five different concentrations of standard substances dissolved in methanol. The resulting curve equations were used for quantitative determination of each phenolic compound. Hydroxybenzoic acids were quantified as gallic acid equivalents (R^2^ = 0.99 with LOD = 0.36 μg/mL and LOQ = 1.44 μg/mL), flavanols as catechin equivalents (R^2^= 0.99 with LOD = 0.18 μg/mL and LOQ = 0.72 μg/mL), and flavonols as rutin equivalents (R^2^= 0.99 with LOD = 0.21 μg/mL and LOQ = 0.84 μg/mL).

### 2.4. In Vitro Antioxidant Activity Analysis

#### 2.4.1. 2,2-Diphenyl-1-picrylhydrazyl (DPPH) Radical Scavenging Capacity

DPPH radical scavenging activity was evaluated as previously described [17].

A 0.1 mM DPPH solution prepared in methanol was mixed 1:1 (*v*/*v*) with the extract at the tested concentrations and incubated for 30 min in the dark at room temperature. Absorbance was recorded at 517 nm and results were expressed as µmol Trolox equivalents per mL extract (µmol TE/mL).

#### 2.4.2. Ferric Reducing Antioxidant Power (FRAP) Assay

The ferric reducing antioxidant power (FRAP) was assessed by monitoring the reduction in the Fe^3+^–TPTZ complex at 593 nm. Values were reported as μmol Trolox equivalents per mL extract (μmol TE/mL) [17].

#### 2.4.3. Hydrogen Peroxide (H_2_O_2_) Scavenging Activity

Hydrogen peroxide scavenging capacity was determined by incubating 1 mL of extract with 2 mL of 40 mM H_2_O_2_ in phosphate buffer (pH 7.4) for 10 min, followed by absorbance reading at 230 nm versus a blank lacking H_2_O_2_. Inhibition was calculated relative to the H_2_O_2_-containing control without extract [18]. Results were presented as μmol Trolox equivalents per mL extract (μmol TE/mL).

#### 2.4.4. Nitric Oxide (NO) Radical Scavenging Assay

Nitric oxide scavenging activity was measured using sodium nitroprusside (10 mM) as an NO generator. The reaction mixture (SNP in buffered saline) was incubated with the extract at 25 °C for 150 min under light; afterwards, equal volumes of sample and Griess reagent were combined and absorbance was measured at 540 nm. Results were expressed as µmol quercetin equivalents per mL extract (µmol QE/mL) [19].

### 2.5. In Vivo Experimental Design

#### 2.5.1. Animal Models

The experimental animals were male Wistar rats (200–250 g). The subjects were obtained from the Center for the Breeding and Use of Laboratory Animals of the “Iuliu Hațieganu” University of Medicine and Pharmacy, Cluj-Napoca, Romania. The animals were housed under standard laboratory conditions with a temperature of 22 ± 2 °C, a 12 h light/dark cycle, 50 ± 10% relative humidity, and access to food and water. Animals were monitored daily for general condition and overt clinical signs throughout the experimental period, and no apparent distress was observed. The experiments were designed in accordance with national and international recommendations for the care and use of laboratory animals. The study protocols were reviewed and approved by the institutional ethics committee under number (373/4 July 2023).

#### 2.5.2. Experimental Protocol

Two experimental approaches were employed to evaluate the therapeutic and prophylactic effects of *L. decidua* needle extract. To investigate the therapeutic potential, acute inflammation was induced on the first day, except for the negative control group. Treatments were administered for seven days, starting on day 2. The animals were randomly allocated into seven experimental groups, each comprising five rats. The first group served as a healthy control, receiving no inflammatory stimulus (Control, C). A second group was assigned to the inflammation model, in which inflammation was induced using turpentine oil (Inflammation, I). Three additional groups received standard anti-inflammatory or antioxidant treatments: one group was administered diclofenac at a dose of 10 mg/kg body weight/day (DICLO), while another received Trolox at 50 mg/kg body weight/day (TROLOX). The remaining three groups were treated with *L. decidua* extract at different concentrations obtained by diluting with distilled water, each rat receiving 1 mL/day of either the 100% extract dilution (1 g:1 mL) (L100), the 50% extract dilution (0.5 g:1 mL) (L50), or the 25% extract dilution (0.25 g:1 mL) (L25).

To emphasize the prophylactic potential of the extracts, animals were randomly assigned to receive *L. decidua* extract in the same three concentrations of 100%, 50%, and 25%, designated as groups pL100, pL50, and pL25, respectively. In this protocol, a Trolox group was added. The prophylaxis was performed by oral gavage from day 1 to day 7. Acute inflammation was induced on day 8. A negative control group, and a one-day inflammation group induced on day 8 were also used.

The experimental model of acute inflammation was obtained by injecting turpentine oil 6 mL/kg body weight into the hind paw of the experimental animal. Treatments were administered by oral gavage by administrating 1 mL/day of the tested product. Rats from the control and inflammation groups received 1 mL/day of tap water.

On day 9 of both therapeutic and prophylaxis protocols, animals were anesthetized with ketamine (60 mg/kg b. w.) and xylazine (15 mg/kg b. w.) administered intraperitoneally, blood samples were collected via retro-orbital puncture, and serum was separated and stored at −20 °C until analysis.

### 2.6. Biochemical and Oxidative Stress Marker Analysis

#### 2.6.1. Total Oxidative Status (TOS)

Serum TOS was measured using Erel’s automated colorimetric method and expressed as µM H_2_O_2_ equivalents/L [20].

#### 2.6.2. Total Antioxidant Capacity (TAC)

Serum TAC levels were determined calorimetrically based on the ability of antioxidants in the analyzed solution to reduce the ABTS radical cation. The results were determined according to the Trolox standard curve. The absorbance was recorded at 660 nm, and the values were expressed in mmol Trolox equivalents per liter (mmol TE/L) [21].

#### 2.6.3. Oxidative Stress Index (OSI)

The OSI was calculated as the ratio of the total oxidant status (TOS) to the total antioxidant capacity (TAC), both expressed in the same units, and multiplied by 100 [22].

#### 2.6.4. 8-Hydroxy-2′-deoxyguanosine (8-OHdG)

Levels of 8-OHdG, a biomarker of oxidative DNA damage, were determined using an ELISA kit (catalog No: ER1487, Wuhan, China), according to the manufacturer’s instructions. Serum samples and standards were added to wells pre-coated with an 8-OHdG conjugate, followed by incubation with a specific antibody. The absorbance was read at 450 nm, and the concentrations were expressed in ng/mL [23].

#### 2.6.5. Advanced Oxidation Protein Products (AOPP)

AOPP concentrations were assessed by a spectrophotometric method based on the oxidation of proteins by chloramine-T, resulting in the formation of dityrosine-containing cross-linked protein products. The serum samples were mixed with potassium iodide and acetic acid, and the absorbance was measured at 340 nm. The results were expressed as μmol/L of chloramine-T equivalents [24].

#### 2.6.6. Malondialdehyde (MDA)

MDA levels, which reflect lipid peroxidation, were determined by the thiobarbituric acid reactive substances (TBARS) method. Serum was mixed with TBARS and incubated in boiling water for 15 min. After cooling, the absorbance was measured at 532 nm and the results were expressed in nmol/mL [25].

#### 2.6.7. Nitric Oxide (NO)

The synthesis of nitric oxide (NO) was indirectly assessed by quantifying total nitrites and nitrates by the Griess reaction. In summary, serum proteins were eliminated through extraction using a 3:1 (*v*/*v*) methanol/diethyl ether solution, the nitrates were converted to nitrites via the addition of vanadium (III) chloride, and subsequently, the Griess reagent was introduced. The absorbance of the sample was measured at 540 nm, and the findings were reported as nitrite μmol/L [26].

#### 2.6.8. Total Thiols (SH)

Total thiol content was determined using Ellman’s reagent (DTNB), which reacts with sulfhydryl groups to form a yellow chromophore measurable at 412 nm. The results were expressed as μmol/L [27].

#### 2.6.9. 3-Nitrotyrosine (3-NT)

Levels of 3-NT, a marker of protein nitration, were evaluated using a competitive ELISA kit (catalog No: EU2560, FineTest, Wuhan, China). The procedure followed the manufacturer’s specifications, and absorbance was measured at 450 nm. Results were expressed in nanomoles per milliliter [28].

### 2.7. Inflammatory Marker Analysis

The anti-inflammatory response was evaluated by quantifying serum NFκB-p65 (ELISA kit catalog No: ER1187, FineTest, Wuhan, China), interleukin-1β (IL-1β) No: ER1094 (Wuhan, China), and interleukin-18 (IL-18) using ELISA kit catalogue No: ER0036 (FineTest, Wuhan, Hubei, China) performed according to the manufacturers’ protocols. For NFκB-p65, the results were expressed in pg/mL. High-sensitivity ELISA kits were employed for IL-1β and IL-18, with absorbance likewise measured at 450 nm, and final cytokine concentrations reported in pg/mL.

### 2.8. Statistical Analysis

Statistical analysis was conducted using SPSS Statistics version 26.0 for Windows (SPSS Inc., Chicago, IL, USA) and R 4.5.1 software (R Foundation for Statistical Computing, Vienna, Austria), with data presented as mean ± standard deviation (SD) for normally distributed variables. Group comparisons were conducted using one-way ANOVA, followed by Bonferroni–Holm post hoc analyses. Correlation analyses were conducted with the Pearson test, and principal component analysis (PCA) was employed to assess the correlations among variables. The threshold for statistical significance was established at *p* < 0.05.

## 3. Results

### 3.1. Phytochemical Analysis

#### 3.1.1. Total Flavonoid Content and Total Polyphenol Content

The results of the total flavonoid content (TFC) and total polyphenol content (TPC) of the *L. decidua* needles extract are presented in Table 1.

#### 3.1.2. Phytochemical Profiling Through HPLC–ESI-MS

**Figure 1:**
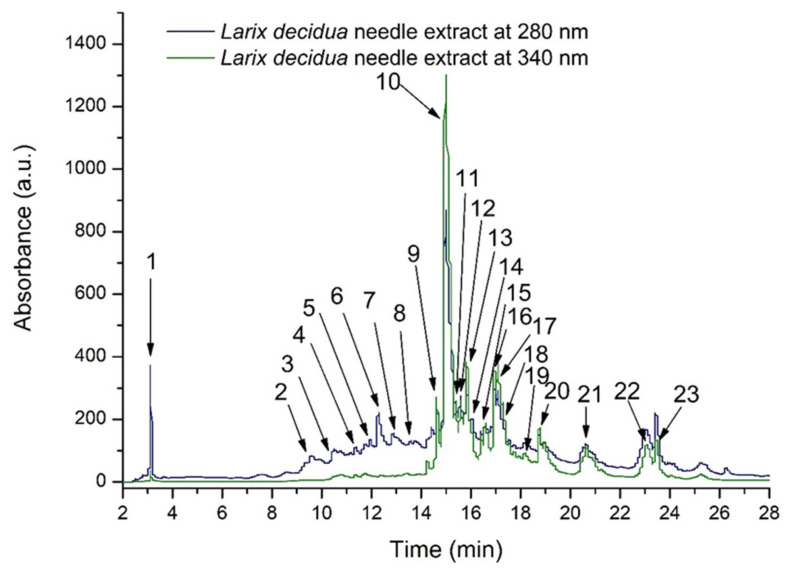
Representative HPLC chromatogram of *Larix decidua* needle extract recorded at 280 and 340 nm. Peaks identification is presented in Table 2.

The chromatographic HPLC fingerprint of *L. decidua* needle extract is presented in Figure 1. The HPLC analysis highlighted a total of 23 compounds, mainly belonging to flavonols (81.57%), flavanols (15.45%), and hydroxybenzoic acids (2.97%) (Table 2).

Among these, kaempferol-rhamnosyl-rhamnoside (803.87 ± 7.53 µg/mL), catechin (140.62 ± 5.53 µg/mL), quercetin-glucoside (162.75 ± 2.63 µg/mL), and quercetin-rhamnoside (154.38 ± 9.00 µg/mL) were the most abundant constituents. The total phenolic content quantified by HPLC was 2600.78 ± 18.94 µg/mL.

### 3.2. In Vitro Antioxidant Activity

The *L. decidua* extract demonstrated relevant antioxidant and radical scavenging potential in all applied assays. Compared to Trolox, *L. decidua* extract exhibited a similar DPPH radical scavenging capacity (*p* > 0.05), a moderate H_2_O_2_ scavenging activity (*p* < 0.01), and a high ferric reducing antioxidant power (*p* < 0.001); *L. decidua* extract NO scavenging activity (*p* < 0.001) was better than that of quercetin (*p* < 0.001). The complete results are presented in Table 3.

### 3.3. In Vivo Antioxidant and Anti-Inflammatory Activity

**Figure 2:**
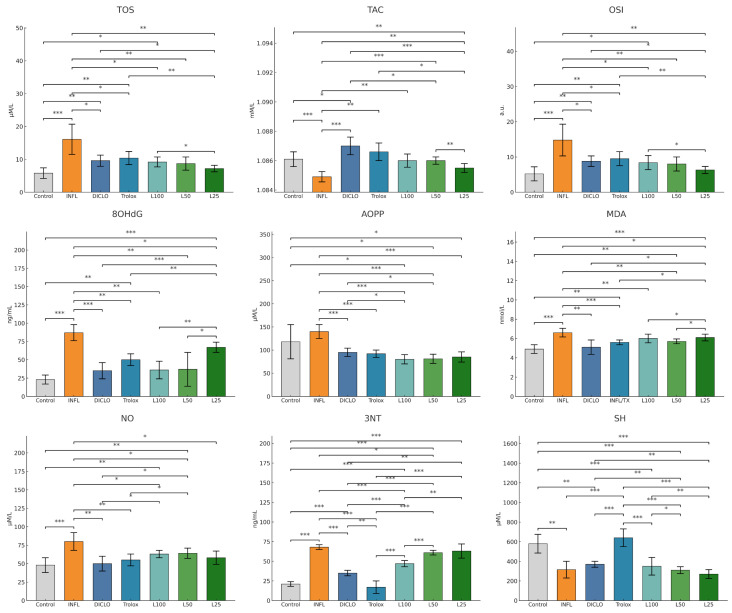
The therapeutic effect of *L. decidua* extracts on oxidative stress parameters. * *p* < 0.05; ** *p* < 0.01; *** *p* < 0.001; TOS—total oxidative status; TAC—total antioxidant capacity; OSI—oxidative stress index; MDA—malondialdehyde; AOPP—advanced oxidation protein products; 8-OHdG—8-hydroxy-2′-deoxyguanosine; NO—nitric oxide; 3NT—3-nitrotyrosine; SH—total thiols; DICLO—diclofenac (10 mg/kg); Trolox—Trolox (50 mg/kg); L100—group treated with *L. decidua* extract 1 g:1 mL; L50—group treated with *L. decidua* extract 0.5 g:1 mL; L25—group treated with *L. decidua* extract 0.25 g:1 mL; INFL—inflammation induced by turpentine oil.

Turpentine oil injections induced an oxidative imbalance and a statistically significant inflammatory response compared to the control group. As shown in Figure 2, after a week, the inflammation produced significant increases in OSI, MDA, TOS, 3-NT, 8-OHdG, AOPP, and NO, accompanied by a marked reduction in TAC and SH levels (all *p* < 0.001 vs. control). Therapeutic administration of *L. decidua* extracts significantly attenuated the oxidative stress parameters in a concentration-dependent manner. L100 demonstrated the strongest effect, restoring MDA, OSI, and 8-OHdG close to control values (*p* < 0.01 vs. INFL). On the other hand, the 50% extract concentration induced moderate improvements (*p* < 0.05 vs. INFL), and (*p* < 0.05 vs. INFL) L25 showed partial efficacy. Diclofenac and Trolox confirmed their reference antioxidant roles, with values overlapping those of L100 in most parameters.

**Figure 3:**
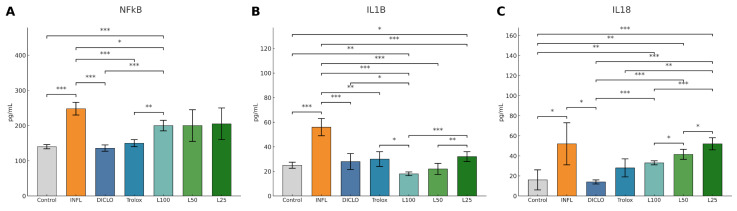
The therapeutic effect of *L. decidua* extracts on inflammatory marker parameters. * *p* < 0.05; ** *p* < 0.01; *** *p* < 0.001; NFkB—nuclear factor kappa B; IL18—interleukin 18; IL1B—interleukin 1 beta; DICLO—diclofenac (10 mg/kg); Trolox—Trolox (50 mg/kg); L100—group treated with *L. decidua* extract 1 g:1 mL; L50—group treated with *L. decidua* extract 0.5 g:1 mL; L25—group treated with *L. decidua* extract 0.25 g:1 mL; INFL—inflammation induced by turpentine oil.

The inflammatory markers tested in vivo and represented in Figure 3, NFκB-p65, IL-1β, and IL-18, were significantly increased in the inflammation group compared to the control group (*p* < 0.01). *L. decidua* extracts produced a dose-dependent suppression of these parameters. L100 exerted the most pronounced effect (*p* < 0.01 vs. INFL), approaching the efficacy of diclofenac and Trolox. L50 also significantly reduced NFκB-p65 and IL-18 (*p* < 0.05 vs. INFL), while (*p* < 0.05 vs. INFL) L25 showed only a partial reduction.

**Figure 4:**
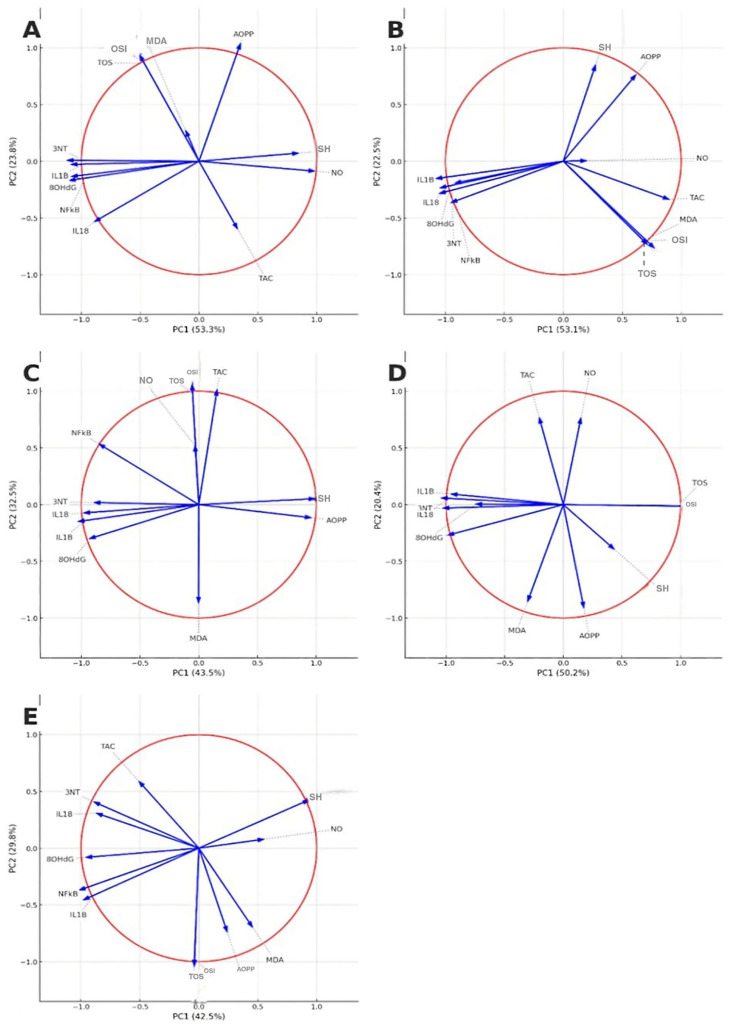
PCA correlation circles of oxidative stress and inflammatory markers in experimental groups. Vectors represent the contribution and correlation of each biomarker with the first two principal components (PC1 and PC2). (**A**) INFL group; (**B**) DICLO group; (**C**) L100—group treated with *L. decidua* extract 1 g:1 mL; (**D**) L50—group treated with *L. decidua* extract 0.5 g:1 mL; (**E**) L25—group treated with *L. decidua* extract 0.25 g:1 mL. The direction and length of vectors indicate the weight of each variable in discriminating between groups. TOS—total oxidative status; TAC—total antioxidant capacity; OSI—oxidative stress index; MD—malondialdehyde; AOPP—advanced oxidation protein products; 8-OHdG—8-hydroxy-2′-deoxyguanosine; NO—nitric oxide; 3-NT—3-nitrotyrosine; SH—total thiols; NFκB—nuclear factor kappa B; IL1B—interleukin-1 beta; IL18—interleukin-18.

The principal component analysis (PCA) was applied to investigate the relationship between oxidative stress and inflammatory biomarkers and to assess the overall effect of the treatment. Sampling adequacy was confirmed (KMO = 0.757), and Bartlett’s test of sphericity was significant (χ^2^ = 515.0, *df* = 66, *p* < 0.001), indicating suitability for factor analysis. Two principal components with eigenvalues > 1 were retained, explaining 72.1% of the total variance (PC1 = 53.2%, PC2 = 18.9%). The rotated component structure revealed that most of the oxidative and inflammatory variables (MDA, AOPP, 8OHdG, 3NT, NO, IL-1β, IL-18, NFκB-p65) loaded on PC1, representing the oxidative–inflammatory axis. PC2 was mainly defined by TOS, OSI, and TAC, reflecting the variability of the oxidant-antioxidant balance. The PCA score plot demonstrated a distinct clustering of animals in the positive control group (INFL) compared to the control group, validating that acute inflammation was characterized by a consistent increase in oxidative and inflammatory markers. The correlation circles highlighted in Figure 4 provided insight into the effect of the treatments.

In the INFL group (A) all markers were projected in similarity, with high loadings on PC1, highlighting their strong interdependence in the inflammatory state. In the diclofenac group (B), the projection of IL-1β, IL-18, and NFκB-p65 was reduced compared to INFL, indicating attenuation of the inflammatory response, although oxidative markers (MDA, AOPP) remained prominent (Figure 4).

Within the L100 group (C), the *L. decidua* extract at the highest tested concentration produced a more balanced distribution of oxidative and inflammatory markers. Therefore, L100 attenuated the oxidative–inflammatory load, approaching the pattern observed with the reference drug. The 50% concentration (D) produced an intermediate repositioning, with partial separation from the inflamed group, indicating a moderate protective effect. The lowest concentration of 25%, group L25 (E), resulted in minor changes, with most markers maintaining a distribution similar to the inflamed profile (Figure 4).

The PCA showed that the distribution of biomarkers varied according to the concentration of *L. decidua* extract. The 100% extract (L100) group clustered closer to the reference treatment (diclofenac), while the L50 and L25 groups showed intermediate profiles between the inflamed and the control animals, indicating a dose-dependent modulation of the oxidative and inflammatory markers.

### 3.4. Prophylactic Protocol

**Figure 5:**
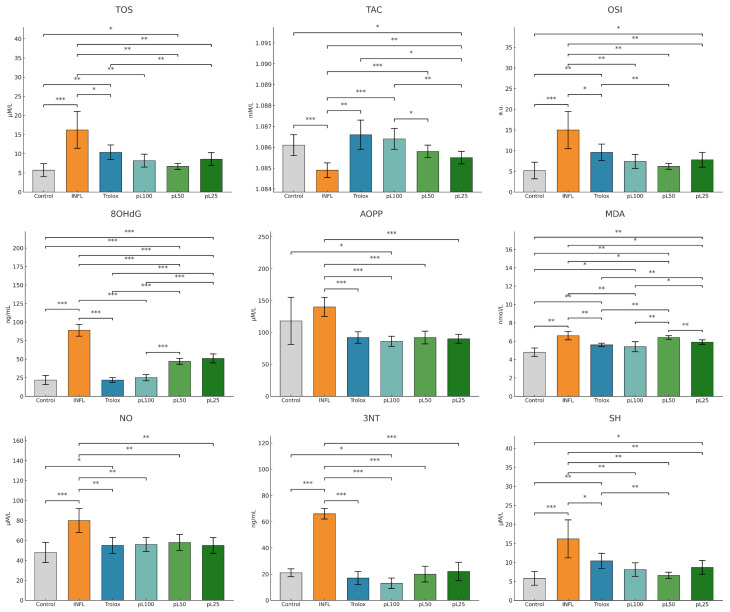
The prophylactic effect of *L. decidua* extracts on oxidative stress parameters. * *p* < 0.05; ** *p* < 0.01; *** *p* < 0.001; TOS—total oxidative status; TAC—total antioxidant capacity; OSI—oxidative stress index; MDA—malondialdehyde; AOPP—advanced oxidation protein products; 8-OHdG—8-hydroxy-2′-deoxyguanosine; NO—nitric oxide; 3NT—3-nitrotyrosine; SH—total thiols; Trolox—Trolox (50 mg/kg); pL100—prophylactic *L. decidua* extract 1 g:1 mL administration; pL50—prophylactic *L. decidua* extract 0.5 g:1 mL administration; pL25—prophylactic *L. decidua* extract 0.25 g:1 mL administration; INFL—inflammation induced by turpentine oil.

*L. decidua* extract administered prophylactically provided protection against oxidative and inflammatory changes. The one-day acute inflammation model obtained by intramuscular injection of turpentine oil significantly increased MDA, TOS, OSI, 3-NT, 8-OHdG, AOPP, and NO, while reducing serum TAC and SH levels (*p* < 0.001 vs. control) (Figure 5).

In Figure 5 it can be seen that prophylactic administration of *L. decidua* extracts attenuated these changes in a concentration-dependent manner. pL100 produced the strongest effect, restoring MDA, TOS, OSI, 3-NT, and 8-OHdG to values significantly lower than those of inflamed rats (*p* < 0.01 vs. INFL), compared to Trolox (*p* > 0.05). pL50 also improved oxidative stress indices, although to a lesser extent (*p* < 0.05 vs. INFL), while (*p* < 0.05 vs. INFL) pL25 showed limited but detectable effects. TAC and thiol groups were significantly preserved in the pL100 group (*p* < 0.01 vs. INFL), with a partial improvement in pL50 (*p* < 0.05 vs. INFL).

**Figure 6:**
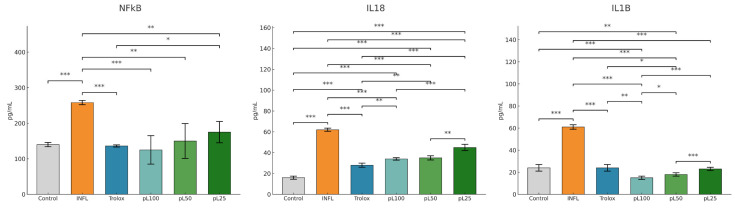
The prophylactic effect of *L. decidua* extracts on inflammatory markers parameters. * *p* < 0.05; ** *p* < 0.01; *** *p* < 0.001; NFkB—nuclear factor kappa B—p65; IL18—interleukin 18; IL1B—interleukin 1 beta; DICLO—diclofenac (10 mg/kg); TROLOX—Trolox (50 mg/kg); pL100—prophylactic *L. decidua* extract 1 g:1 mL administration; pL50—prophylactic *L. decidua* extract 0.5 g:1 mL administration; pL25—prophylactic *L. decidua* extract 0.25 g:1 mL administration; INFL—inflammation induced by turpentine oil.

The impact of the prophylactic administration of *L. decidua* extracts on the inflammatory markers is represented in Figure 6. *L. decidua* extracts significantly prevented NFκB-p65 and IL-18 increase in a dose-dependent manner, with the 100% concentration showing the greatest reduction (*p* < 0.01 vs. INFL). For IL-1β, all concentrations of *L. decidua* extracts induced significant suppression compared to the inflammation group (*p* < 0.05), with the best effect obtained at the 100% concentration.

PCA was run to explore the distribution of oxidative stress and inflammatory markers in the experiment that studied the prophylactic effects of *L. decidua* needle extracts. Sampling adequacy was confirmed (KMO > 0.7; Bartlett’s test of sphericity, *p* < 0.001), and the communalities for all variables exceeded 0.79, indicating that the selected markers were well explained by the retained components. The first principal component (PC1) explained 70.5% of the variance, while the second (PC2) contributed 9.9%, together representing over 80% of the total variance. PC1 was predominantly defined by oxidative stress markers (TOS, OSI, 8-OHdG, 3-NT, AOPP) and inflammatory mediators (IL-1β, IL-18, NFκB-p65), reflecting the oxidative–inflammatory axis. PC2 was mainly characterized by lipid peroxidation in contrast to TAC.

**Figure 7:**
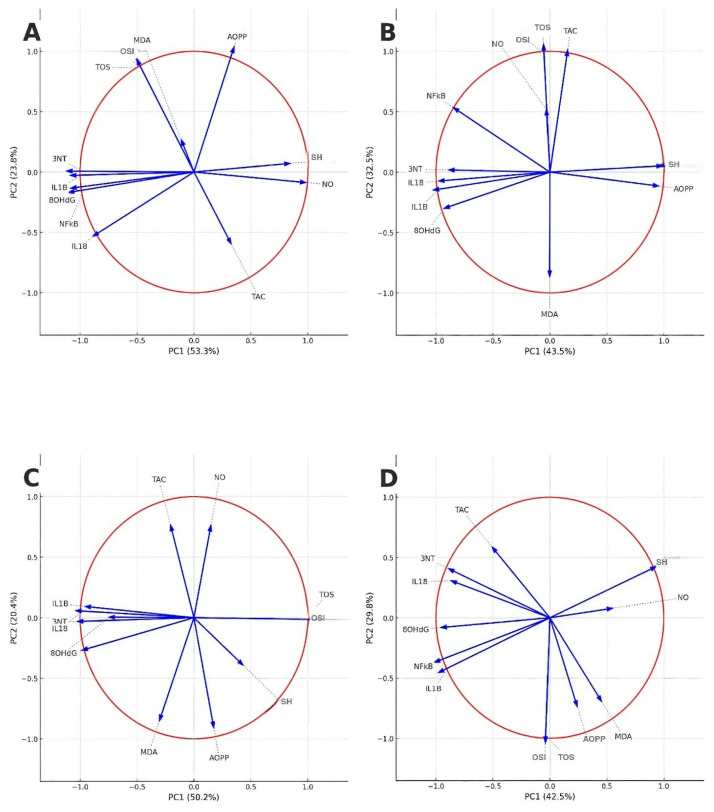
PCA correlation circles of oxidative stress and inflammatory markers in experimental groups. Vectors represent the contribution and correlation of each biomarker with the first two principal components (PC1 and PC2). (**A**) INFL—inflammation group; (**B**) pL100—prophylactic *L. decidua* extract 1 g:1 mL administration; (**C**) pL50—prophylactic *L. decidua* extract 0.5 g:1 mL administration; (**D**) pL25—prophylactic *L. decidua* extract 0.25 g:1 mL administration. The direction and length of vectors indicate the weight of each variable in discriminating between groups. TOS—total oxidative status; TAC—total antioxidant capacity; OSI—oxidative stress index; MD—malondialdehyde; AOPP—advanced oxidation protein products; 8-OHdG—8-hydroxy-2′-deoxyguanosine; NO—nitric oxide; 3-NT—3-nitrotyrosine; SH—total thiols; NFκB—nuclear factor kappa B; IL1B—interleukin-1 beta; IL18—interleukin-18.

The correlation plots in Figure 7 illustrate the prophylactic administration-pendent modulation of these biomarker profiles. In the positive control group with inflammation alone (A), most parameters clustered along PC1, confirming their interdependence during acute inflammation. Prophylactic administration of *L. decidua* extracts modified this distribution in a dose-dependent manner. At the highest concentration (B, pL100), markers were distributed more evenly between PC1 and PC2, with a reduced intensity of clustering, indicating attenuation of the oxidative–inflammatory axis. The middle dose (C, pL50) presented an intermediate pattern, with partial separation from the inflamed profile, while the lowest concentration (D, pL25) produced only limited changes, maintaining a configuration closer to the positive control group in which only inflammation was induced.

## 4. Discussion

Our experimental results demonstrated that *L. decidua* needle extract has significant antioxidant and anti-inflammatory effects.

*L. decidua* phytochemical studies have identified a diverse range of bioactive compounds, including taxifolin, flavonoids, phenolic acids, and monoterpenes such as β-myrcene, all known for their antioxidant and anti-inflammatory effects [29]. The phytochemical analysis and in vitro tests support the hypothesis suggesting that *L. decidua* needle extracts possess antioxidant activity.

The extract scavenged DPPH• radicals almost as efficiently as Trolox, while demonstrating a stronger ferric reducing antioxidant power (FRAP) than Trolox. This suggests that the phenols present *in L. decidua* extracts are potent radical scavengers, even if their overall reducing capacity is slightly lower than that of standard antioxidants [29]. High DPPH• scavenging correlates with increased levels of flavonoids in the extract, especially (+)-catechin, identified as a major constituent of larch needle extracts. Catechins are known for their ability to donate electrons or hydrogen atoms to stabilize free radicals. An example is epigallocatechin gallate, which exhibits exceptionally potent ROS-inhibiting activity due to its multiple hydroxyl groups [30]. Also, the presence of kaempferol glycosides in the extract represents an important factor in the increased capacity to capture H_2_O_2_ and NO [31]. Another important compound contributing to the positive results is taxifolin (dihydroquercetin), a flavonoid present in extracts of *Larix* spp., which is known to have antioxidant properties [32]. The synergistic effects of polyphenol-type compounds identified in larch needle extracts, the flavonols, are an additional explanation for the broad-spectrum in vitro antioxidant effects tests, thus suggesting a possible antioxidant and anti-inflammatory protection in vivo.

The antioxidant activity of *Larix decidua* extract is reflected in its capacity to correct the redox imbalance triggered by acute inflammation. In experimental animals in the INFL group, we observed the expected increase in oxidative stress markers, along with depletion of antioxidant defenses.

In the in vivo turpentine oil-induced experimental model of acute inflammation, TOS and TAC assessed the overall oxidative and antioxidant status, respectively, offering an integrative view of redox homeostasis. OSI, derived from their ratio, reflected the shift toward the oxidative imbalance typical of acute inflammatory states [9]. In the group in which only inflammation was induced, the positive control, the TOS and OSI levels of the experimental animals were higher. This finding aligns with the data from the specialty literature, which shows an increase in these oxidative stress indicators in parallel with the depletion of the antioxidant reserves. Administration of the extract prevented excessive ROS/RNS generation and preserved the antioxidant capacity. In rats treated with *L. decidua* extract, TOS and OSI levels were significantly lower compared to the untreated inflamed rats, reflecting an overall reduction in systemic oxidant load. The prophylactic administration was more efficient than the therapeutic one, suggesting that the presence of exogenous antioxidants can prevent better oxidants accumulation. *L. decidua* extracts increased TAC, and the effect was more pronounced with prophylactic administration. This can be due to the indirect effect of reduction in the oxidants by prophylactic *L. decidua* extract use, and the consequent decrease in the antioxidant consumption.

Hydroxy-2′-deoxyguanosine (8-OHdG) reflected oxidative DNA damage resulting from ROS-mediated guanine oxidation. Its increased levels, often promoted by 3-NT, serve as a marker of DNA injury during acute inflammation [10]. Malondialdehyde (MDA) is a byproduct of the lipid peroxidation process, being an indicator of oxidative damage to cell membranes. Elevated MDA levels correlated with the intensity of inflammation and ROS-induced injury [11]. Treatment with the *L. decidua* extract attenuated these changes. High concentration of *Larix decidua* extract effectively reduced lipid peroxidation, an effect attributable to its rich content of polyphenols (e.g., taxifolin, flavonoids), known for their radical scavenging activity [33]. In our study, prophylaxis with *L. decidua* extract kept markers such as MDA and 8-OHdG lower than in the INFL group and, at the 100% concentration, close to control values. These results indicate that prophylactic administration may prevent lipid peroxidation and oxidative DNA damage better than correcting them.

A significant consequence of oxidative stress is the oxidation of plasma proteins. Advanced oxidation protein products (AOPPs) are markers of protein oxidation that also act as pro-inflammatory mediators. Their accumulation stimulates neutrophil activation and cytokine release, contributing to the perpetuation of inflammation [12]. These data are also validated in our experiment, where we found significantly increased AOPP levels in the group with inflammation. This phenomenon is a result of the activation of neutrophils and the release of myeloperoxidase during acute inflammation, generating chlorinated oxidants that modify proteins. On the other hand, AOPP itself can participate in the amplification of the inflammatory process. It can activate NFκB signaling and stimulate NADPH oxidase, leading to additional ROS production [34]. AOPP levels were also significantly reduced in the extract-treated groups. In the prophylactic plan, by preventing the excessive protein oxidation, the extract not only limits the structural damage to proteins, but can also interrupt the vicious cycle of inflammation: decreasing AOPPs can reduce NFκB-p65 activation and secondary ROS generation [35].

During acute inflammation, nitric oxide (NO) is predominantly produced by iNOS in immune cells and contributes to nitrosative stress. NO levels were significantly increased in rats in the positive control group. During acute inflammation, inducible nitric oxide synthase (iNOS) is upregulated, for example, by the expression of NFκB-p65-driven genes in activated macrophages, causing excessive release of NO. While NO at physiological levels has signaling roles, an overproduction of NO is cytotoxic and contributes to tissue damage and to the maintenance and amplification of the inflammatory process. Excess NO also reacts rapidly with superoxide, another ROS abundant in inflamed tissues, to form peroxynitrite (ONOO^−^), a highly reactive RNS. Peroxynitrite mediates the nitration of protein tyrosine, producing 3-nitrotyrosine (3-NT)—a stable end product and a hallmark of nitrosative stress [36]. In our model, inflammation resulted in a marked increase in 3-NT, indicating substantial peroxynitrite formation and nitrative protein damage. This is consistent with the idea that 3-NT is a reliable biomarker of ROS/RNS imbalance in inflammatory disorders [36]. *L. decidua* extracts attenuated the nitrosative stress. Treated rats showed significantly lower levels than control rats. We attribute this to an inhibition of iNOS expression or activity by the bioactive compounds of the extract. Many polyphenol-rich plant extracts are known to reduce iNOS by modulating inflammatory signaling pathways, such as inhibiting NFκB-p65 [37]. It is plausible that the constituents of the *L. decidua* extract (flavonoids, phenolic acids, etc.) suppressed upstream signals that drive excessive NO synthesis in activated immune cells. NO reduction is beneficial not only in itself (less direct RNS-mediated cytotoxicity), but also because it limits peroxynitrite formation. The *L. decidua* treatment resulted in a significant decrease in 3-nitrotyrosine levels relative to inflammation control. This indicates that protein nitration was reduced, consistent with a decrease in peroxynitrite generation. Mechanistically, the reduction in both superoxide (via antioxidant activity) and NO (via iNOS inhibition) provides a dual blockade of peroxynitrite production, thereby protecting proteins from tyrosine nitration. Notably, the ability of *L. decidua* extract to reduce 3-NT in our study is comparable to what has been observed with potent antioxidants in similar experimental models. For example, in a model of turpentine-induced inflammation, the polyphenol-rich *Artemisia dracunculus* extract reduced 3-NT to a degree comparable to Trolox and even outperformed the allopathic anti-inflammatory drug diclofenac in attenuating protein nitration [38].

In addition, the *L. decidua* extract preserved SH and TAC, key components of the antioxidant defense. The therapeutic administration enhanced the antioxidant status, but did not completely reverse the initial oxidative stress [39,40]. These nuances suggest that although *L. decidua* extract has free radical scavenging properties in both investigated protocols, the antioxidant defense reduction during inflammatory insult can be better prevented than corrected.

Oxidative stress marker results confirm that botanical extracts rich in polyphenols can influence the critical oxidative stress markers (TOS/OSI), inhibit lipids, DNA, protein oxidation, and reduce nitrative stress (NO/3-NT) in inflammatory states, frequently exhibiting efficacy akin to traditional antioxidants or NSAIDs.

The undiluted *L. decidua* extract often restored these markers to normal levels. These results align with traditional uses of European larch for treating wounds and inflammation and support findings from previous in vitro studies [41,42,43]. At the highest concentration, the anti-inflammatory efficacy of *L. decidua* extract approached that of standard treatments with diclofenac and Trolox. This supports the idea that herbal remedies can achieve pharmacological effects comparable to allopathic drugs.

The administration of *Larix decidua* needle extract produced a clear improvement in markers of oxidative and nitrosative stress in both prophylactic and therapeutic treatment protocols. This suggests that *L. decidua* extract helped restore redox balance, either by directly scavenging ROS/RNS or by strengthening the endogenous antioxidant defenses. The rich polyphenol content of the extract is likely responsible, as plant polyphenols can donate electrons to neutralize free radicals and chelate transition metals that catalyze ROS formation.

*L. decidua* is a conifer widely used in herbal remedies of traditional medicine to treat infections, which suggests it has anti-inflammatory properties [44]. Phytochemical studies have identified a diverse range of bioactive compounds, including taxifolin, flavonoids, phenolic acids, and monoterpenes such as β-myrcene, all known for their antioxidant and anti-inflammatory effects [29]. Several studies suggest that extracts of *L. decidua* possess promising anti-inflammatory potential. In vitro tests highlight the property of inhibiting key inflammatory enzymes, as well as their ability to inhibit neutrophil activation [4].

In the present study *L. decidua* extracts decreased key inflammatory biomarkers, including NFκB-p65, IL-1β, and IL-18. Nuclear factor kappa B (NFκB-p65) is a redox-sensitive transcription factor that regulates the expression of key pro-inflammatory mediators [45]. Its activation during oxidative stress enhances cytokine production, making it a central target in anti-inflammatory strategies. Interleukin-1β (IL-1β) and interleukin-18 (IL-18) are upregulated via NLRP3 inflammasome activation in response to ROS and inflammatory signals. IL-1β and IL-18 amplify the inflammatory response, and their inhibition is a hallmark of effective anti-inflammatory interventions [46]. In our acute inflammation model, its activation was significantly attenuated by *L. decidua* extract, particularly at the 100% concentration. This finding is biologically plausible given the extract’s phytochemical profile: flavonoids such as taxifolin can inhibit NFκB-p65 signaling and downstream cytokine production [47,48]. The concomitant reduction in IL-1β and IL-18 is significant because these cytokines are matured via activation of the NLRP3 inflammasome by NFκB-p65 during acute inflammation and amplify the inflammatory cascade. Their inhibition is a hallmark of effective anti-inflammatory intervention [49]. In our study, *L. decidua* extracts reduced IL-1B release across all tested doses, with the best effect at 100%. It also reduced IL-18 in a concentration dependent manner, indicating interference with upstream inflammatory signaling. These effects are consistent with findings for other plants investigated for anti-inflammatory potential. The broad cytokine modulation by *L. decidua* extract suggests a multitarget mechanism involving direct inhibition of inflammatory enzyme systems supported by in vitro data showing COX/5-LOX inhibition by *L. decidua* extract and indirect effects via ROS reduction, thereby dampening redox-sensitive pro-inflammatory pathways [41]. The net in vivo consequence is attenuation of the positive feedback loop between oxidative stress and inflammation, an essential therapeutic goal, since unchecked NFκB-p65 and inflammasome activation can drive acute inflammation toward chronicity and, consequently, chronic pathologies.

In our study, regardless of whether the extract was administered prophylactically or therapeutically, it modulated markers of oxidative stress and markers of inflammation in a beneficial direction. The prophylactic administration likely armed the system with an increased antioxidant capacity, directly neutralized the initial rapid increase in ROS/RNS, and reduced antioxidant consumption, thus preventing the full development of oxidative damage. The therapeutic administration, on the other hand, contributed to the rapid elimination of ongoing ROS/RNS and possibly to the acceleration of the resolution of oxidative damage induced by inflammation. The differences observed between the two protocols were modest, indicating that *L. decidua* extract is effective as both a preventive and an interventional remedy. A similar dual approach was reported by Erhan et al. with a bark extract of *Phellodendron amurense*. In their study, this polyphenol- and alkaloid-rich extract significantly reduced markers of oxidative stress, including NO and 3-NT, and inflammatory mediators [15]. Our results with *L. decidua* align with this paradigm, suggesting that its bioactive constituents may act both prophylactically to build resistance to oxidative damage and therapeutically to attenuate post-insult damage. Such flexibility is valuable for potential translational applications, as it implies that the extract could be used to both prevent oxidative bursts related to inflammation and treat acute inflammatory episodes.

PCA of biochemical markers provides additional information by correlating the effects of the extract with a combined oxidative–inflammatory mechanism. In the PCA score plot, treated samples separated from controls along principal components heavily weighted on both oxidative stress indices and inflammatory mediators. In particular, biomarkers of oxidative damage (e.g., lipid peroxides or ROS levels) clustered together with inflammatory biomarkers (e.g., nitrite or nitric oxide-derived cytokines) on the same principal component, forming an oxidative–inflammatory axis. In other words, PCA analysis revealed that variables such as oxidative markers and pro-inflammatory cytokines share common variance, supporting the idea that oxidative stress and inflammation drive each other [50]. Administration of *L. decidua* extract appears to interfere with this cycle. Animals treated with *L. decidua* extract showed a normalization of both classes of biomarkers, which is reflected by a shift in the entire oxidative–inflammatory cluster towards the control group profile in the PCA biplot. Indeed, multivariate analysis in a related context identified a combined “oxidative stress-inflammation” factor (e.g., co-loading of protein carbonyls or peroxides with *C*-reactive protein and IL-6) as a main component explaining the severity of the disease [51]. In our study, the attenuation of both oxidative stress and inflammatory response by treatment with *L. decidua* extracts highlights the ability of the extract to target both pathophysiological mechanisms. By tandemly reducing ROS generation and inflammatory signaling, the extract effectively shifts the biochemical environment towards homeostasis. This observation supported by PCA-type statistical analysis supports the hypothesis that *L. decidua* needle extract exerts its benefits by modulating the interconnected oxidative and inflammatory pathways, rather than acting on a single one.

From the mechanism of action, the antioxidant and anti-inflammatory effects of *L. decidua* extract are comparable to those of other well-known phytotherapeutics that target the oxidative–inflammatory interconnected effect, such as curcumin, [27] epigallocatechin-3-gallate (EGCG) [52] thymoquinone [20], carnosic acid, and carnosol. These have a catechin structure that confers direct antioxidant effects, scavenging ROS and simultaneously interfering with pro-inflammatory pathways by inhibiting iNOS/COX-2 expression and preventing nuclear translocation of NFκB-p65 in stimulated macrophages [21]. The convergence of antioxidant and anti-inflammatory mechanisms resulting from the administration of derived compounds leads to the effective attenuation of oxidative stress which translates into the attenuation of inflammation and vice versa. *L. decidua* needle extract fits into this therapeutic picture, because its phenolic constituents resemble those of green tea, turmeric, black cumin, or rosemary, managing to produce a dual, antioxidant, and anti-inflammatory protection. Such comparisons not only strengthen the plausibility of our findings but also position *L. decidua* in the pantheon of medicinal plants that modulate redox balance and immune responses in unison. This integrated oxidative–inflammatory modulation has undeniable therapeutic valences, suggesting that *L. decidua* extract could have broad relevance as an adjuvant or natural remedy in inflammatory disorders related to oxidative stress.

Our study presents a number of limitations due to the small sample size, which may increase the risk of type II error and limit the statistical power. Another aspect is the use of a single experimental model of acute inflammation. Although this model is well established for oxidative and inflammatory responses, it does not capture the complexity of chronic or multifactorial inflammatory diseases. Extending the investigation to models of arthritis, colitis or metabolic inflammation would provide a more complete understanding of the therapeutic relevance of the extract. Another limitation is the short intervention period. Chronic dosing regimens were not evaluated, leaving unanswered questions about long-term efficacy and safety, which will be the subject of future research.

From a phytochemical point of view, the extract was characterized using HPLC-DAD-ESI-MS, which provided important qualitative and quantitative information. However, batch-to-batch variability in phytochemical composition due to environmental factors or the season in which the plants were harvested was not evaluated. The extracts were obtained by a single extraction method, which may not fully capture all classes of bioactive compounds, such as volatile terpenes or polysaccharides. Although we identified the major phenolic compounds, additional metabolomic and pharmacokinetic analyses are needed to fully characterize the compounds or their metabolites that reach the systemic circulation and contribute to the in vivo effects.

Another important limitation is that safety/toxicology evaluation criteria such as impact on biochemical, hematological parameters, and histopathological evaluation were not included in the present work. However, data on preparations derived from larch are cited in the literature; these suggest a good safety profile. A food-grade bark extract of *L. decidua* administered orally to rats at a dose of 2000 mg/kg body weight was not associated with clinical signs of toxicity or mortality, and the reported LD50 was >5000 mg/kg body weight [42]. Furthermore, supplementation with *L. decidua* sawdust was investigated as a feed additive in dairy cows with monitoring of blood parameters and was reported to have good palatability [4]. In addition, *L. decidua* arabinogalactan has been used as a dietary fiber/feed additive and has been described as having a good safety profile [5]. Another analysis evaluated taxifolin-rich *L. decidua* extract as a novel food ingredient for addition to beverages, yogurt, and confectionery, identifying a NOAEL of 1500 mg/kg body weight in a 90-day rat study [53]. Taken together, these data support the general feasibility of *L. decidua*-derived ingredients and key constituents (e.g., taxifolin) supporting a good toxicity profile. However, a comprehensive toxicological characterization of the needle extract present—especially under chronic dosing conditions—is needed.

Future studies should aim to address these limitations and expand the translational potential of *L. decidua* needle extract. It is important to note that the dual prophylactic and therapeutic efficacy demonstrated in this study highlights *L. decidua* needle extract as a candidate for both preventive nutraceutical strategies and adjuvant therapy in inflammatory diseases. This bidirectional applicability is relatively uncommon among plant extracts and deserves further exploration. By systematically addressing these future directions, *L. decidua* could become a standardized, evidence-based phytopharmaceutical for inflammatory disorders.

## 5. Conclusions

This study provides preclinical evidence that an ethanolic *L. decidua* needle extract obtained by a green repercolation approach exerts antioxidant and anti-inflammatory effects in a turpentine oil-induced model of acute sterile inflammation in rats. Both therapeutic and prophylactic administration attenuated systemic oxidative–nitrosative stress, as reflected by reductions in TOS, OSI, AOPP, MDA, 8-OHdG, NO, and 3-NT, while supporting endogenous antioxidant defenses (TAC and SH). In parallel, the extract reduced key inflammatory mediators (NFκB, IL-1β, and IL-18) in a concentration-dependent manner, with the highest tested concentration (L100) showing effects comparable to the reference treatments for several endpoints.

Comprehensive phytochemical profiling revealed a polyphenol-rich composition, supporting a multi-target modulation of the oxidative–inflammatory axis and consistent clustering patterns in PCA. From a translational perspective, these findings support further exploration of standardized *L. decidua* needle preparations as a nutraceutical/functional ingredient candidate and as a potential adjunct to dietary strategies targeting oxidative stress-driven inflammatory processes, particularly in preventive settings. However, the present results derive from an acute preclinical model using a crude extract, and therefore clinical efficacy cannot be inferred. Future work should address standardization using quantitative marker compounds, bioavailability, and comprehensive safety assessment, and should validate efficacy in chronic and disease-relevant inflammatory models prior to considering human studies.

## Figures and Tables

**Table 1:** Flavonoid and polyphenol content.

Conifer Extract	TFC (mg QE/g)	TPC (mg GAE/g)
*L. decidua*	2.07 ± 0.22	3.442 ± 0.22

QE = quercetin equivalents; GAE = gallic acid equivalents.

**Table 2:** HPLC–DAD–ESI–MS profile of phenolic compounds in *Larix decidua* extract.

Peak	Rt (min)	UV λmax (nm)	[M + H]^+^ (*m*/*z*)	Compound	Subclass	Content (µg/mL)
1	3.12	275	155	2,3-Dihydroxybenzoic acid	Hydroxybenzoic acid	60.40 ± 2.84
2	9.55	280	307	Gallocatechin	Flavanol	52.30 ± 5.57
3	10.48	275	155	2,4-Dihydroxybenzoic acid	Hydroxybenzoic acid	16.89 ± 2.67
4	11.38	280	579	Procyanidin dimer B3	Flavanol	51.02 ± 2.18
5	11.94	280	579	Procyanidin dimer B1	Flavanol	47.47 ± 0.33
6	12.25	280	291	Catechin	Flavanol	140.62 ± 5.53
7	12.83	280	579	Procyanidin dimer B2	Flavanol	73.75 ± 2.55
8	13.51	280	291	Epicatechin	Flavanol	36.73 ± 2.23
9	14.58	358,255	521,317	Isorhamnetin-acetyl-glucoside	Flavonol	91.84 ± 2.31
10	14.96	350,260	579,287	Kaempferol-rhamnosyl-rhamnoside	Flavonol	803.87 ± 7.53
11	15.4	350,260	433,287	Kaempferol-rhamnoside	Flavonol	81.54 ± 1.18
12	15.61	350,250	465,319	Myricetin-rhamnoside	Flavonol	86.74 ± 4.12
13	15.86	360,255	465,303	Quercetin-glucoside	Flavonol	162.75 ± 2.63
14	16.09	358,255	493,317	Isorhamnetin-glucuronide	Flavonol	69.47 ± 1.97
15	16.6	358,255	595,317	Isorhamnetin-arabinosyl-rhamnoside	Flavonol	102.68 ± 0.31
16	16.97	350,260	449,287	Kaempferol-glucoside	Flavonol	111.97 ± 1.89
17	17.12	360,255	449,303	Quercetin-rhamnoside	Flavonol	154.38 ± 9.00
18	17.31	358,255	479,317	Isorhamnetin-glucoside	Flavonol	95.07 ± 3.78
19	18.19	350,260	419,287	Kaempferol-arabinoside	Flavonol	62.07 ± 0.95
20	18.79	350,260	491,287	Kaempferol-acetyl-glucoside	Flavonol	66.21 ± 0.60
21	20.64	358,255	625,317	Isorhamnetin-neohesperidoside	Flavonol	50.86 ± 1.16
22	23.09	350,260	287	Kaempferol	Flavonol	105.71 ± 0.71
23	23.44	360,270	305	Taxifolin	Flavonol	76.44 ± 0.79

**Table 3:** Antioxidant and anti-inflammatory activity in vitro of *L decidua* extract.

Sample/Standard	DPPH (µg TE/mL)	H_2_O_2_ (µg TE/mL)	FRAP (µg TE/mL)	NO (µg QE/mL)
*L. decidua*	10.01 ± 0.25	74.90 ± 0.84	126.69 ± 0.65	119.01 ± 0.37
Trolox	11.20 ± 0.45	24.23 ± 1.53	19.97 ± 1.10	-
Quercetin	-	-	-	20.58 ± 2.68

TE = Trolox equivalents; QE = quercetin equivalents; DPPH = 2,2-diphenyl-1-picrylhydrazyl radical scavenging; H_2_O_2_ = hydrogen peroxide scavenging activity; FRAP = ferric reducing antioxidant power; NO = nitric oxide scavenging activity.

## Data Availability

The original contributions presented in this study are included in the article. Further inquiries can be directed to the corresponding author.
